# Use of central nervous system drugs in combination with selective serotonin reuptake inhibitor treatment: A Bayesian screening study for risk of suicidal behavior

**DOI:** 10.3389/fpsyt.2022.1012650

**Published:** 2022-11-09

**Authors:** Tyra Lagerberg, Arvid Sjölander, Robert D. Gibbons, Patrick D. Quinn, Brian M. D’Onofrio, Clara Hellner, Paul Lichtenstein, Seena Fazel, Zheng Chang

**Affiliations:** ^1^Department of Medical Epidemiology and Biostatistics, Karolinska Institutet, Stockholm, Sweden; ^2^Departments of Medicine and Public Health Sciences, Center for Health Statistics, University of Chicago, Chicago, IL, United States; ^3^Department of Applied Health Science, School of Public Health, Indiana University, Bloomington, IN, United States; ^4^Department of Psychological and Brain Sciences, Indiana University, Bloomington, IN, United States; ^5^Department of Clinical Neuroscience, Center for Psychiatry Research, Karolinska Institutet, Stockholm, Sweden; ^6^Stockholm Health Care Services, Stockholm County Council, Stockholm, Sweden; ^7^Department of Psychiatry, Warneford Hospital, University of Oxford, Oxford, United Kingdom

**Keywords:** screening study, selective serotonin reuptake inhibitor, suicidal behavior, central nervous system drugs, Bayesian

## Abstract

**Background:**

Using other central nervous system (CNS) medications in combination with selective serotonin reuptake inhibitor (SSRI) treatment is common. Despite this, there is limited evidence on the impact on suicidal behavior of combining specific medications. We aim to provide evidence on signals for suicidal behavior risk when initiating CNS drugs during and outside of SSRI treatment.

**Materials and methods:**

Using a linkage of Swedish national registers, we identified a national cohort of SSRI users aged 6–59 years residing in Sweden 2006–2013. We used a two-stage Bayesian Poisson model to estimate the incidence rate ratio (IRR) of suicidal behavior in periods up to 90 days before and after a CNS drug initiation during SSRI treatment, while accounting for multiple testing. For comparison, and to assess whether there were interactions between SSRIs and other CNS drugs, we also estimated the IRR of initiating the CNS drug without SSRI treatment.

**Results:**

We identified 53 common CNS drugs initiated during SSRI treatment, dispensed to 262,721 individuals. We found 20 CNS drugs with statistically significant IRRs. Of these, two showed a greater risk of suicidal behavior after versus before initiating the CNS drug (alprazolam, IRR = 1.39; flunitrazepam, IRR = 1.83). We found several novel signals of drugs that were statistically significantly associated with a reduction in the suicidal behavior risk. We did not find evidence of harmful interactions between SSRIs and the selected CNS drugs.

**Conclusion:**

Several of the detected signals for reduced risk correspond to drugs where there is previous evidence of benefit for antidepressant augmentation (e.g., olanzapine, quetiapine, lithium, buspirone, and mirtazapine). Novel signals of reduced suicidal behavior risk, including for lamotrigine, valproic acid, risperidone, and melatonin, warrant further investigation.

## Introduction

Antidepressant medications are the principal pharmacological treatments for mood and anxiety disorders, and selective serotonin reuptake inhibitors (SSRIs) are the most common antidepressant class in many countries (1). Meanwhile, concurrent treatment with two or more CNS drugs is becoming increasingly prevalent in several Western countries, among adults (2–4) as well as among the young (5, 6). For example, US psychiatrist visits where patients were prescribed two or more CNS drugs increased from 43 to 60% between 1996–7 and 2005–6 (3), with combinations including antidepressants constituting the most common type of co-prescription.

Central nervous system (CNS) co-medication with SSRIs may be motivated by clinical need. Though SSRIs have shown efficacy in treating core depressive symptoms (7), around 50–60% of patients do not respond to treatment with the first SSRI they are prescribed (8). Antidepressant switching or augmentation with additional antidepressants or other CNS drugs could be required in such cases (3, 9), Mood disorders also show a high degree of comorbidity with other psychiatric disorders, meaning that prescription of additional CNS drugs concurrently with SSRIs may be warranted based on the comorbidity profile of the individual (10). For example, a US study found that patients were treated with more than one drug in about 69% of hospitalizations for major depression (11).

However, drug-drug interactions (DDIs) that may be either harmful or beneficial can occur from co-administering SSRIs with other CNS drugs. For example, SSRIs have been shown to inhibit the clearance of different CNS drugs, including antipsychotics (12). Despite this, the majority of evidence and guidelines regarding SSRI treatment relate to individual medications, meaning that clinicians have relatively little guidance on the risks and benefits of specific drug combinations (13). The risk of suicide attempts or deaths (“suicidal behavior”) is a particularly important consideration as part of the safety and efficacy profile of antidepressant treatment (14). With recently developed pharmacoepidemiological methods (15, 16) and population-wide register data, we have the possibility to more comprehensively investigate the risk of suicidal behavior associated with concurrent use of other CNS medications during SSRI treatment in real-world data.

We therefore assessed whether adding additional CNS drugs during SSRI treatment was associated with the risk of suicidal behavior using a data-driven screening approach. We also compared the risk of suicidal behavior when initiating CNS drugs with and without SSRI treatment. The aim was to identify signals that will help guide future research on the efficacy and safety, in terms of suicidal behavior, of adding specific CNS drugs to SSRI treatment.

## Materials and methods

### Data sources

We linked information from different Swedish national registers using unique personal identification numbers (17). Prescription information was obtained from the Swedish Prescribed Drug Register, which has information on all dispensed drugs in Sweden since July 2005 (18). The National Patient Register (NPR), supplied records of inpatient care since 1973 and specialist outpatient care since 2001 (19). We extracted information on dates and causes of death from The Cause of Death Register (20); demographic information from the Total Population Register (21); and emigration data from the Migration Register (21). It is not necessary to obtain informed consent for register-based studies in Sweden (22). Our study has been approved by the Regional Ethics Committee (Stockholm, Sweden).

### Cohort

Our study design is an extension of the screening approach developed by Gibbons et al. to identify drugs associated with risk of suicidal events (15). The current study aims to explore risk of suicidal behavior associated with adding non-SSRI CNS drugs to continuous SSRI treatment, and to evaluate whether there are drug-drug interactions between SSRIs and other CNS drugs. For this purpose, we started by defining a cohort of SSRI users, which included individuals prescribed with an SSRI (N06AB) between the ages 6 to 65 years in Sweden from July 2006 to December 2013. Supplementary Table 1 shows the types of SSRIs sold in Sweden during the study period. Treatment periods with the SSRIs were defined as follows: a treatment period started at the dispensation date of a prescription. Two dispensations falling within 120 days (4 months) of each other were considered to belong to the same treatment period (23). 30 days were added to the end of the last prescription in a treatment period.

**FIGURE 1:**
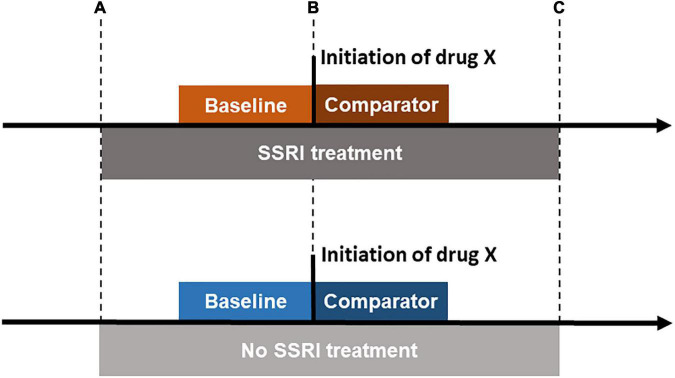
Illustration of central nervous system (CNS) drug initiation during and outside of selective serotonin reuptake inhibitor (SSRI) treatment. The time period between points **(A,B)** and between **(B,C)** is required to be ≥ 30 days. For CNS drug initiations during SSRI treatment, the CNS drug initiation had to occur ≥ 30 days since the first SSRI prescription in the treatment period and at or before the last prescription in the continuous treatment period (the end of the treatment period is defined by adding 30 days to the date of the last prescription). For CNS drug initiations outside of SSRI treatment, the CNS drug initiation had to occur ≥ 30 days since the end of the last SSRI treatment period and at least 30 days before the first prescription in the next SSRI treatment period (if applicable).

We then identified all occasions when individuals initiated another prescribed CNS drug during SSRI treatment periods. We considered CNS drugs in any of the following ATC classes: N02A (opioids), N03A (antiepileptics), N05A (antipsychotics), N05B (anxiolytics), N05C (hypnotics and sedatives), N06A (antidepressants), N06B (psychostimulants), and N07B (drugs used in addictive disorders) (5). Figure 1 illustrates the study design. We identified initiation of a CNS medication as the first dispensed prescription after at least a 365-day period free of that medication, and at least 30 days after the start of the SSRI treatment period (in order to ensure that the suicide risk after initiation with the added drug was not driven by the initiation of the SSRI treatment itself). Each individual could contribute more than one occasions of initiating another CNS drug, as long as they were at least 365 days apart. We included CNS drugs that were initiated on at least 1,000 occasions during treatment with any SSRI, and where at least 20 outcome events (defined below) were recorded in the 90-day period before or after initiation across individuals (see Supplementary Table 2 for the included drug ATC codes and names). This was a pragmatic decision made to ensure acceptable power for each initiating CNS drug, adapted from Gibbons et al. (15). We also identified all instances where any individual initiated the same set of CNS drugs outside of SSRI treatment.

### Measures

#### Exposure

The exposure of interest was initiation of specific CNS drugs during or outside SSRI treatment. The baseline period lasted up to 90 days prior to the drug initiation; the comparison period lasted up to 90 days following initiation (Figure 1). We required all of the baseline and comparator periods to occur either during or outside SSRI treatment, depending on whether CNS initiation during or outside SSRI treatment was considered. The minimum duration of the baseline period was 30 days (see Figure 1). Follow-up in the comparator period was further censored at the date of the first recorded emigration or death, whichever occurred first within 90 days after the initiation (if applicable).

#### Outcome

The outcome was suicidal behavior. This included outpatient attendance or inpatient admission for suicide attempts and deaths from suicide. We included both events where intent was known and unknown (ICD-10 codes X60-X84 and Y10-Y34, respectively) (24). In sensitivity analyses, we have used (1) only events of known intent and (2) only suicide attempts (as we cannot capture deaths happening before the initiation of the additional CNS drug).

#### Analysis

We used a two stage Bayesian Poisson regression model to estimate Incidence Rate Ratios (IRRs) and credible intervals (CrI) while account for multiple testing (25, 26). CrIs are used in Bayesian statistics: with a 95% CrI, you can say with 95% probability that the true parameter value lies within the CrI (27). In the first stage model, the incidence rate of suicidal behavior in the period after initiating a specific CNS drug (comparator period) was compared to the incidence rate in the period before initiating the drug (baseline period) in the same group of individuals, with comparisons made within each combination of the initiating CNS drug and the baseline treatment (no or any SSRI). The first stage model was adjusted for baseline treatment with any SSRI (yes/no), sex, and age categories (6–24, 25–34, 35–44, 45–54, and 55–65 years). In order to account for multiple testing, we included a second-stage model containing a variable that reflects biological similarity of the included CNS drugs. This variable was the third level of the ATC codes (e.g., N02A). All estimates were “shrunk” toward each other within the third level ATC code–that is, all estimates within the ATC groupings were pulled toward each other (25). See the Supplementary material for details on the model.

We estimated IRRs for CNS drug initiations both during and outside of continuous SSRI treatment. We then took the ratio between them to test if there was any interaction between the specific CNS drug and SSRI treatment for the risk of suicidal behavior. Estimating the ratio of ratios is a way to test whether there is a difference between the IRR for CNS drug initiation during and outside SSRI treatment. A ratio of ratios that is over 1 shows that the IRR of suicidal behavior when a CNS drug is initiated during SSRI treatment is greater than the IRR when a CNS drug is initiated outside of SSRI treatment.

In secondary analyses, we investigated the risk during baseline treatment with specific SSRI types, where the comparisons were made within the type of initiating drug for baseline treatment with a given SSRI type. We also examined the risk of initiating CNS drugs during treatment with any SSRI in males and females separately.

A number of sensitivity analyses were conducted: first, restricting the analysis to individuals aged above 17 years (the determinants of suicidality during SSRI treatment have been found to differ between children/adolescents and adults) (28); second, using only suicidal behavior events of known intent as the outcome; third, including only attempted suicides in the outcome measure; fourth, excluding the date on which the additional prescription was prescribed from analyses (to avoid exposure misclassification when the event happened on the day of prescription); and finally, running the main analysis using a frequentist Poisson regression model without borrowing information across ATC groups.

The data management was carried out in SAS version 9.4. Statistical analyses and figures were generated using R version 3.6.3.

## Results

We identified 53 non-SSRI CNS drugs that were initiated at sufficient frequency during treatment with any SSRI, dispensed to 262,721 individuals (66.4% female, Table 1). We further identified 2,447,617 individuals with CNS drug initiations of the selected 53 drugs outside of any SSRI treatment. For most SSRIs, the majority of individuals with non-SSRI CNS initiations were in middle age (45–54 or 55–65 years old), except for fluoxetine treatment (a majority aged 6–24 years). CNS drug initiation during fluvoxamine treatment was very uncommon, occurring in only 407 individuals–CNS drug initiation during fluvoxamine treatment was therefore not included in the analyses stratifying on specific SSRI types. Supplementary Table 3 shows the number of events during baseline and comparator periods for each of the initiating CNS drugs during any or no SSRI treatment.

**TABLE 1:** Number of individuals initiating another central nervous system (CNS) drugs during selective serotonin reuptake inhibitors (SSRI) treatments^a^.

	Overall	Females	Age group^b^				
			6–24 years	25–34 years	35–44 years	45–54 years	55–65 years
**SSRI treatment**							
Any SSRI	262,721	174,366(66.37%)	34,425(13.1%)	47,213(17.97%)	62,423(23.76%)	68,509(26.08%)	74,442(28.34%)
Fluoxetine	31,464	23,559(74.88%)	7,674(24.39%)	6,542(20.79%)	7,347(23.35%)	6,698(21.29%)	5,735(18.23%)
Citalopram	94,045	62,786(66.76%)	6,409(6.81%)	13,545(14.4%)	21,117(22.45%)	26,037(27.69%)	33,651(35.78%)
Paroxetine	16,188	10,081(62.27%)	1,019(6.29%)	2,719(16.8%)	3,983(24.6%)	4,924(30.42%)	5,214(32.21%)
Sertraline	89,827	59,162(65.86%)	15,068(16.77%)	17,424(19.4%)	21,331(23.75%)	21,535(23.97%)	20,948(23.32%)
Fluvoxamine	407	226(55.53%)	36(8.85%)	67(16.46%)	95(23.34%)	119(29.24%)	120(29.48%)
Escitalopram	38,795	24,562(63.31%)	4,168(10.74%)	7,477(19.27%)	9,624(24.81%)	10,344(26.66%)	9,962(25.68%)
No SSRI treatment	2,447,617	1,306,501(53.38%)	394,221(16.11%)	428,621(17.51%)	548,693(22.42%)	624,982(25.53%)	752,215(30.73%)

^a^Individuals could contribute to more than one type of SSRI. ^b^Individuals could contribute to more than one age category.

**FIGURE 2:**
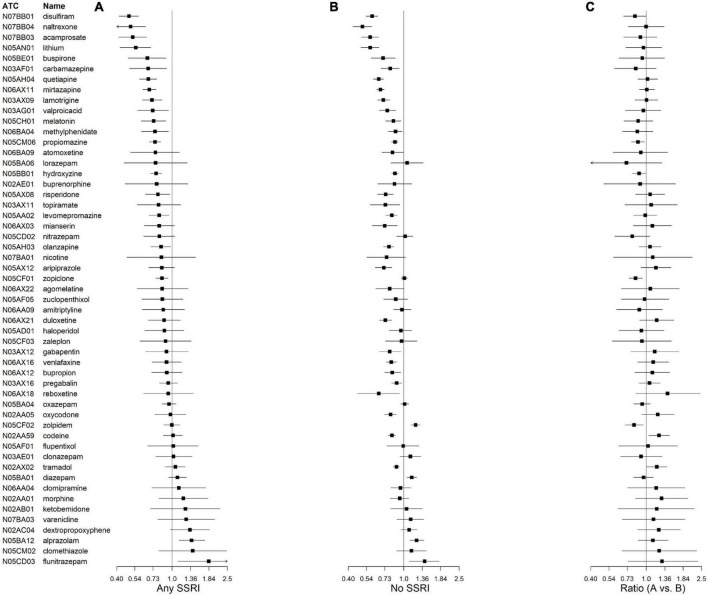
Incidence rate ratios (IRRs) and credible intervals of suicidal behavior associated with central nervous system (CNS) drug initiation during treatment with **(A)** and without **(B)** any selective serotonin reuptake inhibitor (SSRI). Ratio of IRRs in **(A)** vs **(B)** are also presented **(C)**. N02AA59 represents codeine combinations excluding psycholeptics.

Figure 2 shows the IRRs of CNS drug initiation during treatment with any SSRI. Two drugs showed a statistically significantly greater risk of suicide after versus before initiating a CNS drug (alprazolam, IRR = 1.39, 95% CrI = 1.13, 1.71; flunitrazepam, IRR = 1.83, 95% CrI = 1.11, 3.07; Supplementary Table 4). Eighteen drugs showed a statistically significantly reduced risk. Those with the greatest risk reduction were disulfiram (IRR = 0.49, 95% CrI = 0.42, 0.58), naltrexone (IRR = 0.50, 95% CrI = 0.39, 0.65), and acamprosate (IRR = 0.52, 95% CrI = 0.41, 0.66).

Six CNS drugs had significantly different IRRs during baseline treatment with any versus no SSRI (Figure 2; Supplementary Table 4). Four had a lower IRR during SSRI treatment compared to no SSRI treatment: hydroxyzine (IRR ratio = 0.88, 95% CrI = 0.79, 0.99), zopiclone (IRR ratio = 0.84, 95% CrI = 0.75, 0.94), zolpidem (IRR ratio = 0.82, 95% CrI = 0.71, 0.95), and propiomazine (IRR ratio = 0.88, 95% CrI = 0.78, 0.98). Two had a higher IRR during no SSRI treatment: codeine combinations excluding psycholeptics (IRR ratio = 1.24, 95% CrI = 1.05, 1.46), and tramadol (IRR ratio = 1.19, 95% CrI = 1.01, 1.41). Initiation of either of the latter two drugs during treatment with any SSRI showed no association with suicidal behavior.

**FIGURE 3:**
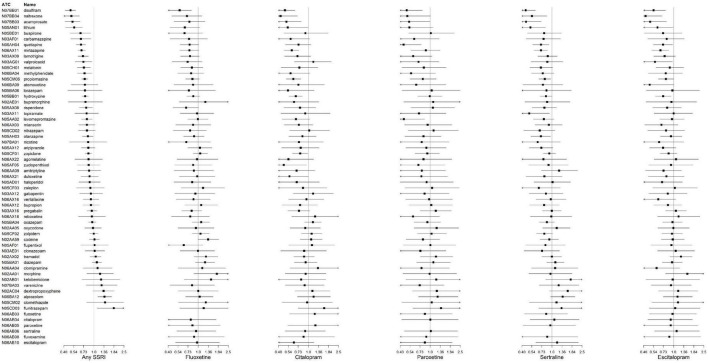
Incidence rate ratios (IRRs) and credible intervals of suicidal behavior associated with central nervous system (CNS) drug initiation during treatment with specific selective serotonin reuptake inhibitors (SSRIs). N02AA59 represents codeine combinations excluding psycholeptics.

When examining the associations during treatment with specific SSRI types (Figure 3), all the statistically significant IRRs showed a reduced risk of suicidal behavior after versus before initiation of the additional CNS drug (Supplementary Table 5). When examining the associations by sex (Supplementary Figure 1), the pattern of IRRs was similar in the overall cohort and females. In males, all statistically significant IRRs showed reduced risk of suicidal behavior after CNS drug initiations (Supplementary Table 6).

Finally, our sensitivity analyses–where only individuals aged above 17 years were included (N initiators during any SSRI treatment = 258,208, Supplementary Figure 2); where using only suicidal behavior of known intent (Supplementary Figure 3); where using only suicide attempts (Supplementary Figure 4); where excluding the date on which the additional CNS drug (Supplementary Figure 5); and where using a frequentist Poisson regression model (Supplementary Figure 6)–all showed similar patterns of results to the main analysis.

## Discussion

In this register-based study, we have screened for the risk of suicidal behavior when non-SSRI CNS drugs are added to SSRI treatment. The majority of CNS drugs were associated with reduced risk of suicidal behavior, and we identified several novel signals for drugs of potential use for reducing suicidal behavior risk during SSRI treatment. We did not find evidence of harmful DDIs, in terms of suicidal behavior risk, from co-administering CNS drugs with SSRIs.

Eighteen drugs were associated with clear reductions in the risk of suicidal behavior when initiated during SSRI treatment, as indicated by statistical significance. This could reflect beneficial effects of treatment augmentation, or appropriate treatment of comorbidity. Regarding treatment augmentation, around 50–60% of patients do not respond to initial monotherapy with an antidepressant, and it may be necessary to add further medications (9). Four antipsychotics have been approved by the FDA for augmentation of antidepressants in cases of treatment-resistant depression: brexpiprazole, aripiprazole, olanzapine when combined with fluoxetine, and quetiapine XR (9). Of those FDA-approved medications that were included in this analysis, olanzapine and quetiapine had a statistically significant reduced risk of suicidal behavior post-initiation (IRR = 0.83 and 0.67, respectively), and aripiprazole showed a null association.

Further medications, including liothyronine, lithium, buspirone, mirtazapine, and bupropion have shown efficacy in non-responders to antidepressant monotherapy (29–33), though it should be noted that the evidence for mirtazapine is somewhat conflicting–further research is necessary (33, 34). Out of these drugs, all but liothyronine were included in the present study, and all but bupropion had statistically significantly lowered risk of suicidal behavior in periods after versus before initiating the drug during antidepressant treatment. The fact that our model identifies drugs that are currently approved–or that have been identified as possible candidates–for antidepressant augmentation gives some reassurance regarding the validity of our model, though augmentation of antidepressant effect may not imply a reduction in suicidal behavior risk. We identified a number of additional drugs with statistically significantly lowered risk during any SSRI treatment, including lamotrigine, valproic acid, risperidone, and melatonin. These novel signals could be further investigated to assess whether they are suitable to combine with SSRIs in terms of lowering the risk of suicidal behavior.

However, it is possible that we see a statistically significant association with reduced risk of suicidal behavior for some of the drugs because adding them to SSRI treatment reflects appropriate treatment of comorbidity. For example, it is notable that all included drugs used to treat alcohol dependence (disulfiram, acamprosate, and naltrexone) are associated with substantially reduced risks of suicidal behavior in the period after versus before initiation of the drugs during treatment with any SSRI. This corresponds with prior epidemiological studies that have found these medications to be associated with reduced risk of suicidal behavior in substance use disorders (35), and in released prisoners (36). These drugs were also found to be associated with statistically significantly reduced risks in the screening study by Gibbons et al. (15). Addiction disorders are highly correlated with suicidal behavior (37). This suggests that appropriate pharmacological treatment of primary and comorbid addiction disorders may be paramount in reducing suicide risk, regardless of whether the drug is administered during or outside of any SSRI treatment. A further possibility is that, if initiation with the additional CNS drug is indicated by a heightened risk of suicide, part of the apparent reduction in risk after initiation is an artifact of the process of selection into treatment (38).

We identified only two CNS drugs associated with statistically significantly increased risk of suicidal behavior when initiated during SSRI treatment. Both are benzodiazepines (alprazolam, IRR = 1.39; and flunitrazepam, IRR = 1.83). Alprazolam was found to be the drug associated with the greatest risk increase in the screening study by Gibbons et al. (15). Findings that benzodiazepines carry risks to patients have led to de-registrations or restrictions of the use of these drugs in several markets. Benzodiazepine treatment has been found to be associated with an increased risk of suicidal behavior in both observational studies (15) and clinical trials (39). Use of these drugs in combination with SSRIs requires attention and further research.

We have not found evidence of harmful interactions between SSRIs and other CNS drugs. Out of the drugs where there was a statistically significant different effect estimate of initiation during SSRI treatment versus outside of SSRI treatment, all but two were associated with a lower risk of suicidal behavior during SSRI treatment. The exceptions (codeine combinations excluding psycholeptics and tramadol) have null associations with suicidal behavior when initiated during SSRI treatment. However, this could be because individuals who are under SSRI treatment are already under greater clinical monitoring.

We did not find different patterns of results for initiation of drugs during SSRI treatment when considering strata by different types of SSRIs or sex. However, these stratified analyses were restricted by limited sample size, meaning we could not draw strong conclusions regarding possible effect modification.

We found similar patterns of results to the main analysis when restricting the analysis to individuals aged over 17 years. Additional sensitivity analyses indicated that the results are relatively robust to different measurement definitions.

### Strengths and limitations

The main strength of our study is that it is based on population registers, ensuring complete coverage of the individuals who received CNS medications no matter the comorbidity profile or sociodemographic background. Another is the application of a novel approach for multiple testing adjustments while screening a large number of drug combinations (25, 26). However, there are several limitations. First, our data do not allow us to determine whether the initiation of additional CNS drugs represents medication augmentation, medication switching, or co-medication due to psychiatric comorbidities. We do not have information on indications for the prescriptions, and so cannot account for selection by diagnoses or diagnosis severity into different types of treatments. Second, we have not accounted for other drugs taken in addition to SSRIs and the selected CNS drugs. It is possible that certain drugs are systematically given in combinations with other drugs that affect the risk and safety profile, although we cannot identify any consistent patterns of co-administration from the literature. Third, it is possible that further factors may influence the risk before and after initiation which we cannot account for. For example, initiation of another CNS drug is a possible indicator of greater clinical monitoring or additional psychological treatment. Fourth, the selection into treatment by an outcome could also contribute to biasing the risk after initiation downward. Fifth, our results derive from Sweden and are not necessarily generalizable to other countries, though many of our detected signals correspond to existing evidence using other designs. Finally, our analysis does not allow us to infer causality–further research investigating medication signals of interest is recommended.

## Conclusion

Our study found that a number of CNS drugs were associated with a reduced risk of suicidal behavior when initiated during SSRI treatment, and two that were associated with increased risk, notably alprazolam. We did not find evidence of harmful drug-drug interactions between the selected CNS drugs and SSRIs. Several of the signals we detect correspond to prior evidence on successful antidepressant augmentation strategies, while the novel signals of reduced risk of suicidal behavior warrant further investigation.

## Data availability statement

The data analyzed in this study is subject to the following licenses/restrictions: The data used is Swedish register data, which is not available for sharing due to privacy considerations. Requests to access these datasets should be directed to Socialstyrelsen, socialstyrelsen@socialstyrelsen.se.

## Ethics statement

The studies involving human participants were reviewed and approved by Regional Ethics Committee (Stockholm, Sweden; decision number 2013/862-31/5). Written informed consent from the participants’ legal guardian/next of kin was not required to participate in this study in accordance with the national legislation and the institutional requirements.

## Author contributions

TL had full access to the data in the study and takes responsibility for the integrity of the data and the accuracy of the data analysis. TL drafted the manuscript, with critical revisions from all authors. TL, ZC, AS, and RG were all involved in the conception and design of the study. TL and AS performed the statistical analyses. All authors contributed to the interpretation of data and agreed to the final version to be published.
