# Supplementary material for: Use of central nervous system drugs in combination with selective serotonin reuptake inhibitor treatment: A Bayesian screening study for risk of suicidal behavior
Source: Front Psychiatry. 2022 Nov 9;13:1012650. doi: 10.3389/fpsyt.2022.1012650 (PMC9682954; doi:10.3389/fpsyt.2022.1012650)
Supplement: Supplementary file 1 [file Data_Sheet_1.DOCX]

**Supplementary material**

**Contents**

[**Table S1. SSRIs prescribed in Sweden during the study period** 2](#_Toc110531785)

[**Table S2. Included CNS drugs** 3](#_Toc110531786)

[**Table S3. Frequency of add-on drug instances and suicide events by baseline treatment with no or any SSRI** 4](#_Toc110531787)

[**Table S4. Statistically significant IRRs for the risk of suicidal behavior associated with CNS drug initiation during treatment with any SSRI or outside of SSRI treatment** 7](#_Toc110531788)

[**Table S5. Statistically significant IRRs for the risk of suicidal behavior associated with CNS drug initiation during treatment with specific SSRIs** 8](#_Toc110531789)

[**Table S6. Statistically significant IRRs for the risk of suicidal behavior associated with CNS drug initiation during any SSRI treatment, by sex** 9](#_Toc110531790)

[**eMethods** 10](#_Toc110531791)

[**Figure S1. IRRs and credible intervals of suicidal behavior from CNS drug initiation during treatment with any SSRI, by sex.** 13](#_Toc110531792)

[**Figure S2. IRRs and credible intervals of suicidal behavior from CNS drug initiation during treatment with any SSRI and outside of SSRI treatment among individuals aged >17 years.** 14](#_Toc110531793)

[**Figure S3. IRRs and credible intervals of suicidal behavior from CNS drug initiation during treatment with any SSRI and outside of SSRI treatment, considering only suicidal behavior of known intent as the outcome** 15](#_Toc110531794)

[**Figure S4. IRRs and credible intervals of suicidal attempts from CNS drug initiation during treatment with any SSRI and outside of SSRI treatment, when only suicide attempts are included in the outcome definition.** 16](#_Toc110531795)

[**Figure S5. IRRs and credible intervals of suicidal behavior from CNS drug initiation during treatment with any SSRI and outside of SSRI treatment, excluding the date on which the additional CNS drug was initiated** 17](#_Toc110531796)

[**Figure S6. IRRs and credible intervals of suicidal behavior from CNS drug initiation during treatment with any SSRI and outside of SSRI treatment in the main analysis when using a frequentist Poisson regression model.** 18](#_Toc110531797)

| **Table S1. SSRIs prescribed in Sweden during the study period** | |
| --- | --- |
| **ATC** | **Name** |
| N06AB03 | Fluoxetine |
| N06AB04 | Citalopram |
| N06AB05 | Paroxetine |
| N06AB06 | Sertraline |
| N06AB08 | Fluvoxamine |
| N06AB10 | Escitalopram |

| **Table S2. Included CNS drugs** | |
| --- | --- |
| **ATC** | **Name** |
| N02AA01 | morphine |
| N02AA05 | oxycodone |
| N02AA59 | codeine, combinations excl. psycholeptics |
| N02AB01 | ketobemidone |
| N02AC04 | dextropropoxyphene |
| N02AE01 | buprenorphine |
| N02AX02 | tramadol |
| N03AE01 | clonazepam |
| N03AF01 | carbamazepine |
| N03AG01 | valproic acid |
| N03AX09 | lamotrigine |
| N03AX11 | topiramate |
| N03AX12 | gabapentin |
| N03AX16 | pregabalin |
| N05AA02 | levomepromazine |
| N05AD01 | haloperidol |
| N05AF01 | flupentixol |
| N05AF05 | zuclopenthixol |
| N05AH03 | olanzapine |
| N05AH04 | quetiapine |
| N05AN01 | lithium |
| N05AX08 | risperidone |
| N05AX12 | aripiprazole |
| N05BA01 | diazepam |
| N05BA04 | oxazepam |
| N05BA06 | lorazepam |
| N05BA12 | alprazolam |
| N05BB01 | hydroxyzine |
| N05BE01 | buspirone |
| N05CD02 | nitrazepam |
| N05CD03 | flunitrazepam |
| N05CF01 | zopiclone |
| N05CF02 | zolpidem |
| N05CF03 | zaleplon |
| N05CH01 | melatonin |
| N05CM02 | clomethiazole |
| N05CM06 | propiomazine |
| N06AA04 | clomipramine |
| N06AA09 | amitriptyline |
| N06AX03 | mianserin |
| N06AX11 | mirtazapine |
| N06AX12 | bupropion |
| N06AX16 | venlafaxine |
| N06AX18 | reboxetine |
| N06AX21 | duloxetine |
| N06AX22 | agomelatine |
| N06BA04 | methylphenidate |
| N06BA09 | atomoxetine |
| N07BA01 | nicotine |
| N07BA03 | varenicline |
| N07BB01 | disulfiram |
| N07BB03 | acamprosate |
| N07BB04 | naltrexone |

# **Table S3. Frequency of add-on drug instances and suicide events by baseline treatment with no or any SSRI**

| **CNS drug** |  | **No SSRI** | **Any SSRI** |
| --- | --- | --- | --- |
| N02AA01 | No. individuals | 88877 | 5530 |
|  | Events baseline | 336 | 41 |
|  | Events comparator | 305 | 48 |
| N02AA05 | No. individuals | 251847 | 13380 |
|  | Events baseline | 971 | 126 |
|  | Events comparator | 772 | 118 |
| N02AA59 | No. individuals | 1000740 | 44314 |
|  | Events baseline | 2482 | 306 |
|  | Events comparator | 2034 | 301 |
| N02AB01 | No. individuals | 29187 | 2118 |
|  | Events baseline | 105 | 20 |
|  | Events comparator | 109 | 25 |
| N02AC04 | No. individuals | 198480 | 9591 |
|  | Events baseline | 411 | 59 |
|  | Events comparator | 448 | 79 |
| N02AE01 | No. individuals | 28878 | 3145 |
|  | Events baseline | 103 | 30 |
|  | Events comparator | 88 | 22 |
| N02AX02 | No. individuals | 797738 | 37859 |
|  | Events baseline | 3004 | 294 |
|  | Events comparator | 2653 | 300 |
| N03AE01 | No. individuals | 11868 | 1914 |
|  | Events baseline | 236 | 86 |
|  | Events comparator | 264 | 87 |
| N03AF01 | No. individuals | 29409 | 2911 |
|  | Events baseline | 374 | 101 |
|  | Events comparator | 299 | 66 |
| N03AG01 | No. individuals | 23234 | 3382 |
|  | Events baseline | 473 | 143 |
|  | Events comparator | 363 | 101 |
| N03AX09 | No. individuals | 39863 | 9414 |
|  | Events baseline | 903 | 370 |
|  | Events comparator | 654 | 257 |
| N03AX11 | No. individuals | 9150 | 1555 |
|  | Events baseline | 148 | 66 |
|  | Events comparator | 109 | 51 |
| N03AX12 | No. individuals | 57330 | 4844 |
|  | Events baseline | 254 | 57 |
|  | Events comparator | 200 | 51 |
| N03AX16 | No. individuals | 91708 | 14986 |
|  | Events baseline | 1121 | 362 |
|  | Events comparator | 998 | 333 |
| N05AA02 | No. individuals | 23414 | 5274 |
|  | Events baseline | 905 | 317 |
|  | Events comparator | 739 | 248 |
| N05AD01 | No. individuals | 10474 | 1756 |
|  | Events baseline | 228 | 75 |
|  | Events comparator | 207 | 64 |
| N05AF01 | No. individuals | 7568 | 2134 |
|  | Events baseline | 107 | 39 |
|  | Events comparator | 106 | 40 |
| N05AF05 | No. individuals | 7152 | 1221 |
|  | Events baseline | 209 | 69 |
|  | Events comparator | 182 | 58 |
| N05AH03 | No. individuals | 39272 | 8200 |
|  | Events baseline | 1152 | 334 |
|  | Events comparator | 895 | 274 |
| N05AH04 | No. individuals | 37246 | 10772 |
|  | Events baseline | 1367 | 483 |
|  | Events comparator | 905 | 317 |
| N05AN01 | No. individuals | 12877 | 2739 |
|  | Events baseline | 468 | 164 |
|  | Events comparator | 270 | 85 |
| N05AX08 | No. individuals | 24257 | 5899 |
|  | Events baseline | 522 | 200 |
|  | Events comparator | 382 | 158 |
| N05AX12 | No. individuals | 18401 | 5319 |
|  | Events baseline | 495 | 195 |
|  | Events comparator | 354 | 160 |
| N05BA01 | No. individuals | 184198 | 23464 |
|  | Events baseline | 1206 | 366 |
|  | Events comparator | 1369 | 391 |
| N05BA04 | No. individuals | 248028 | 44439 |
|  | Events baseline | 1650 | 562 |
|  | Events comparator | 1651 | 520 |
| N05BA06 | No. individuals | 6998 | 1036 |
|  | Events baseline | 108 | 32 |
|  | Events comparator | 112 | 23 |
| N05BA12 | No. individuals | 43274 | 9710 |
|  | Events baseline | 483 | 152 |
|  | Events comparator | 593 | 208 |
| N05BB01 | No. individuals | 473920 | 63047 |
|  | Events baseline | 2660 | 978 |
|  | Events comparator | 2277 | 730 |
| N05BE01 | No. individuals | 9500 | 4221 |
|  | Events baseline | 231 | 92 |
|  | Events comparator | 165 | 59 |
| N05CD02 | No. individuals | 23259 | 3781 |
|  | Events baseline | 425 | 126 |
|  | Events comparator | 431 | 100 |
| N05CD03 | No. individuals | 18673 | 2056 |
|  | Events baseline | 102 | 18 |
|  | Events comparator | 144 | 36 |
| N05CF01 | No. individuals | 435969 | 51101 |
|  | Events baseline | 2627 | 837 |
|  | Events comparator | 2616 | 694 |
| N05CF02 | No. individuals | 356070 | 36919 |
|  | Events baseline | 1228 | 445 |
|  | Events comparator | 1480 | 430 |
| N05CF03 | No. individuals | 15116 | 2327 |
|  | Events baseline | 113 | 42 |
|  | Events comparator | 108 | 36 |
| N05CH01 | No. individuals | 68959 | 9126 |
|  | Events baseline | 523 | 223 |
|  | Events comparator | 437 | 159 |
| N05CM02 | No. individuals | 5159 | 1126 |
|  | Events baseline | 123 | 20 |
|  | Events comparator | 135 | 28 |
| N05CM06 | No. individuals | 336184 | 48694 |
|  | Events baseline | 2838 | 974 |
|  | Events comparator | 2421 | 729 |
| N06AA04 | No. individuals | 16939 | 1528 |
|  | Events baseline | 294 | 34 |
|  | Events comparator | 285 | 36 |
| N06AA09 | No. individuals | 156233 | 9847 |
|  | Events baseline | 374 | 62 |
|  | Events comparator | 364 | 51 |
| N06AX03 | No. individuals | 17516 | 8117 |
|  | Events baseline | 221 | 138 |
|  | Events comparator | 162 | 112 |
| N06AX11 | No. individuals | 165601 | 38891 |
|  | Events baseline | 2513 | 757 |
|  | Events comparator | 1698 | 518 |
| N06AX12 | No. individuals | 66788 | 9446 |
|  | Events baseline | 460 | 122 |
|  | Events comparator | 388 | 104 |
| N06AX16 | No. individuals | 85354 | 7854 |
|  | Events baseline | 1262 | 152 |
|  | Events comparator | 1086 | 122 |
| N06AX18 | No. individuals | 5366 | 3096 |
|  | Events baseline | 77 | 46 |
|  | Events comparator | 51 | 42 |
| N06AX21 | No. individuals | 72152 | 5500 |
|  | Events baseline | 915 | 121 |
|  | Events comparator | 697 | 96 |
| N06AX22 | No. individuals | 9373 | 2011 |
|  | Events baseline | 152 | 43 |
|  | Events comparator | 123 | 35 |
| N06BA04 | No. individuals | 91864 | 8087 |
|  | Events baseline | 636 | 183 |
|  | Events comparator | 552 | 133 |
| N06BA09 | No. individuals | 28261 | 2286 |
|  | Events baseline | 261 | 55 |
|  | Events comparator | 217 | 40 |
| N07BA01 | No. individuals | 19515 | 2191 |
|  | Events baseline | 81 | 21 |
|  | Events comparator | 60 | 18 |
| N07BA03 | No. individuals | 110353 | 6890 |
|  | Events baseline | 134 | 23 |
|  | Events comparator | 152 | 31 |
| N07BB01 | No. individuals | 48008 | 9070 |
|  | Events baseline | 1117 | 468 |
|  | Events comparator | 661 | 221 |
| N07BB03 | No. individuals | 23291 | 4898 |
|  | Events baseline | 515 | 201 |
|  | Events comparator | 296 | 102 |
| N07BB04 | No. individuals | 18999 | 3882 |
|  | Events baseline | 475 | 176 |
|  | Events comparator | 240 | 85 |

# **Table S4. Statistically significant IRRs for the risk of suicidal behavior associated with CNS drug initiation during treatment with any SSRI or outside of SSRI treatment**

|  |  | **Type of treatment** | |  |
| --- | --- | --- | --- | --- |
| **CNS initiation ATC** | **Name** | **Any SSRI**  **(IRR, 95% CrI)** | **No SSRI**  **(IRR, 95%CrI)** | **Ratio**  **(any SSRI vs. no SSRI)** |
| N02AA05 | oxycodone | 0.97 (0.75, 1.25) | 0.80 (0.73, 0.88)* | 1.21 (0.93, 1.58) |
| N02AA59 | codeine, combinations excl. psycholeptics | 1.02 (0.87, 1.19) | 0.82 (0.77, 0.87)* | 1.24 (1.05, 1.46)* |
| N02AX02 | tramadol | 1.05 (0.90, 1.23) | 0.88 (0.84, 0.93)* | 1.19 (1.01, 1.41)* |
| N03AF01 | carbamazepine | 0.67 (0.50, 0.92)* | 0.80 (0.69, 0.93)* | 0.84 (0.60, 1.18) |
| N03AG01 | valproic acid | 0.73 (0.57, 0.94)* | 0.76 (0.67, 0.88)* | 0.96 (0.72, 1.26) |
| N03AX09 | lamotrigine | 0.72 (0.62, 0.85)* | 0.72 (0.65, 0.79)* | 1.01 (0.83, 1.22) |
| N03AX11 | topiramate | 0.80 (0.56, 1.14) | 0.73 (0.57, 0.94)* | 1.09 (0.71, 1.67) |
| N03AX12 | gabapentin | 0.92 (0.64, 1.32) | 0.79 (0.66, 0.95)* | 1.16 (0.78, 1.73) |
| N03AX16 | pregabalin | 0.94 (0.81, 1.09) | 0.89 (0.82, 0.97)* | 1.06 (0.89, 1.25) |
| N05AA02 | levomepromazine | 0.81 (0.69, 0.96)* | 0.82 (0.74, 0.90)* | 0.99 (0.82, 1.20) |
| N05AH03 | olanzapine | 0.83 (0.71, 0.97)* | 0.78 (0.72, 0.85)* | 1.06 (0.89, 1.27) |
| N05AH04 | quetiapine | 0.67 (0.58, 0.77)* | 0.66 (0.60, 0.72)* | 1.02 (0.86, 1.21) |
| N05AN01 | lithium | 0.55 (0.42, 0.71)* | 0.57 (0.49, 0.66)* | 0.96 (0.71, 1.29) |
| N05AX08 | risperidone | 0.79 (0.64, 0.97)* | 0.74 (0.65, 0.84)* | 1.07 (0.84, 1.38) |
| N05AX12 | aripiprazole | 0.84 (0.69, 1.03) | 0.72 (0.62, 0.82)* | 1.17 (0.92, 1.50) |
| N05BA01 | diazepam | 1.10 (0.96, 1.26) | 1.15 (1.06, 1.24)* | 0.95 (0.82, 1.12) |
| N05BA12 | alprazolam | 1.39 (1.13, 1.71)* | 1.25 (1.10, 1.40)* | 1.12 (0.88, 1.43) |
| N05BB01 | hydroxyzine | 0.77 (0.70, 0.84)* | 0.87 (0.82, 0.92)* | 0.88 (0.79, 0.99)* |
| N05BE01 | buspirone | 0.66 (0.48, 0.91)* | 0.72 (0.59, 0.87)* | 0.93 (0.64, 1.35) |
| N05CD03 | flunitrazepam | 1.83 (1.11, 3.07)* | 1.41 (1.10, 1.82)* | 1.30 (0.74, 2.30) |
| N05CF01 | zopiclone | 0.84 (0.76, 0.93)* | 1.00 (0.95, 1.06) | 0.84 (0.75, 0.94)* |
| N05CF02 | zolpidem | 0.99 (0.87, 1.13) | 1.21 (1.13, 1.31)* | 0.82 (0.71, 0.95)* |
| N05CH01 | melatonin | 0.73 (0.60, 0.90)* | 0.84 (0.74, 0.96)* | 0.87 (0.69, 1.11) |
| N05CM06 | propiomazine | 0.75 (0.69, 0.83)* | 0.86 (0.82, 0.91)* | 0.88 (0.78, 0.98)* |
| N06AX03 | mianserin | 0.81 (0.64, 1.04) | 0.73 (0.60, 0.89)* | 1.11 (0.81, 1.52) |
| N06AX11 | mirtazapine | 0.69 (0.62, 0.77)* | 0.68 (0.64, 0.73)* | 1.01 (0.89, 1.15) |
| N06AX12 | bupropion | 0.92 (0.71, 1.19) | 0.84 (0.73, 0.95)* | 1.10 (0.83, 1.47) |
| N06AX16 | venlafaxine | 0.92 (0.73, 1.16) | 0.82 (0.75, 0.88)* | 1.12 (0.88, 1.44) |
| N06AX18 | reboxetine | 0.95 (0.64, 1.40) | 0.66 (0.47, 0.93)* | 1.43 (0.85, 2.43) |
| N06AX21 | duloxetine | 0.88 (0.68, 1.14) | 0.74 (0.67, 0.81)* | 1.19 (0.90, 1.57) |
| N06BA04 | methylphenidate | 0.76 (0.60, 0.94)* | 0.87 (0.78, 0.97)* | 0.87 (0.68, 1.11) |
| N06BA09 | atomoxetine | 0.76 (0.51, 1.14) | 0.84 (0.70, 0.99)* | 0.91 (0.59, 1.41) |
| N07BB01 | disulfiram | 0.49 (0.42, 0.58)* | 0.59 (0.54, 0.65)* | 0.83 (0.69, 1.00) |
| N07BB03 | acamprosate | 0.52 (0.41, 0.66)* | 0.57 (0.50, 0.66)* | 0.91 (0.68, 1.20) |
| N07BB04 | naltrexone | 0.50 (0.39, 0.65)* | 0.50 (0.43, 0.59)* | 1.00 (0.74, 1.34) |

Inclusion in table: significance of either IRR, or of ratio.

# **Table S5. Statistically significant IRRs for the risk of suicidal behavior associated with CNS drug initiation during treatment with specific SSRIs**

|  |  | **Type of SSRI treatment** | | | | |
| --- | --- | --- | --- | --- | --- | --- |
| **CNS initiation ATC** | **Name** | **Fluoxetine**  **(IRR, 95% CrI)** | **Citalopram**  **(IRR, 95% CrI)** | **Paroxetine**  **(IRR, 95% CrI)** | **Sertraline**  **(IRR, 95% CrI)** | **Escitalopram**  **(IRR, 95% CrI)** |
| N03AG01 | valproic acid | 0.72 (0.45, 1.13) | 1.16 (0.66, 2.02) | 0.71 (0.35, 1.34) | 0.86 (0.54, 1.38) | 0.57 (0.34, 0.93)* |
| N03AX11 | topiramate | 0.95 (0.58, 1.60) | 0.92 (0.44, 1.98) | 0.71 (0.31, 1.66) | 0.51 (0.26, 0.96)* | 1.00 (0.54, 1.87) |
| N05AA02 | levomepromazine | 0.99 (0.73, 1.33) | 0.68 (0.45, 1.02) | 0.44 (0.24, 0.84)* | 0.78 (0.56, 1.08) | 0.95 (0.65, 1.34) |
| N05AF05 | zuclopenthixol | 0.86 (0.48, 1.50) | 0.47 (0.23, 0.98)* | 0.69 (0.30, 1.59) | 0.91 (0.51, 1.64) | 0.93 (0.48, 1.77) |
| N05AH03 | olanzapine | 0.88 (0.64, 1.20) | 0.83 (0.56, 1.23) | 0.82 (0.47, 1.44) | 0.71 (0.53, 0.95)* | 0.92 (0.67, 1.25) |
| N05AH04 | quetiapine | 0.77 (0.57, 1.02) | 0.73 (0.48, 1.10) | 0.45 (0.26, 0.77)* | 0.71 (0.55, 0.90)* | 0.72 (0.53, 0.97)* |
| N05AN01 | lithium | 0.67 (0.42, 1.03) | 0.53 (0.28, 0.98)* | 0.53 (0.26, 1.09) | 0.51 (0.33, 0.80)* | 0.61 (0.38, 0.96)* |
| N05BB01 | hydroxyzine | 0.86 (0.71, 1.05) | 0.68 (0.55, 0.83)* | 0.99 (0.69, 1.41) | 0.83 (0.69, 1.00)* | 0.73 (0.57, 0.94)* |
| N05CF01 | zopiclone | 1.06 (0.84, 1.32) | 0.78 (0.62, 0.97)* | 0.92 (0.62, 1.33) | 0.93 (0.77, 1.11) | 0.86 (0.67, 1.10) |
| N05CM06 | propiomazine | 0.83 (0.68, 1.02) | 0.63 (0.51, 0.79)* | 0.80 (0.53, 1.19) | 0.81 (0.67, 0.96)* | 0.79 (0.62, 1.00) |
| N06AX11 | mirtazapine | 0.76 (0.57, 1.02) | 0.60 (0.48, 0.75)* | 0.88 (0.57, 1.34) | 0.72 (0.58, 0.87)* | 0.66 (0.51, 0.87)* |
| N06AX21 | duloxetine | 0.98 (0.56, 1.64) | 0.56 (0.32, 0.99)* | 0.77 (0.36, 1.59) | 0.79 (0.46, 1.33) | 0.80 (0.47, 1.37) |
| N07BB01 | disulfiram | 0.57 (0.40, 0.82)* | 0.49 (0.36, 0.67)* | 0.49 (0.30, 0.78)* | 0.45 (0.34, 0.60)* | 0.55 (0.38, 0.77)* |
| N07BB03 | acamprosate | 0.77 (0.48, 1.22) | 0.51 (0.33, 0.79)* | 0.53 (0.28, 1.03) | 0.46 (0.29, 0.73)* | 0.48 (0.28, 0.82)* |
| N07BB04 | naltrexone | 0.71 (0.44, 1.15) | 0.43 (0.26, 0.71)* | 0.50 (0.24, 1.03) | 0.55 (0.34, 0.85)* | 0.43 (0.26, 0.72)* |

Inclusion in table: significance of either IRR, or of ratio.

# **Table S6. Statistically significant IRRs for the risk of suicidal behavior associated with CNS drug initiation during any SSRI treatment, by sex**

|  |  | **Sex** | |  |
| --- | --- | --- | --- | --- |
| **CNS initiation ATC** | **Name** | **Males**  **(IRR, 95% CrI)** | **Females**  **(IRR, 95% CrI)** | **Ratio**  **(females vs males)** |
| N03AF01 | carbamazepine | 0.60 (0.39, 0.94)* | 0.75 (0.50, 1.14) | 1.25 (0.68, 2.27) |
| N03AG01 | valproic acid | 0.77 (0.47, 1.24) | 0.72 (0.54, 0.96)* | 0.94 (0.54, 1.64) |
| N03AX09 | lamotrigine | 0.73 (0.50, 1.05) | 0.73 (0.60, 0.86)* | 1.00 (0.67, 1.50) |
| N05AA02 | levomepromazine | 0.77 (0.56, 1.08) | 0.81 (0.67, 0.98)* | 1.05 (0.71, 1.54) |
| N05AH04 | quetiapine | 0.69 (0.51, 0.93)* | 0.67 (0.57, 0.78)* | 0.97 (0.69, 1.37) |
| N05AN01 | lithium | 0.38 (0.22, 0.64)* | 0.61 (0.46, 0.81)* | 1.60 (0.89, 2.94) |
| N05AX08 | risperidone | 0.91 (0.63, 1.30) | 0.74 (0.58, 0.94)* | 0.81 (0.53, 1.26) |
| N05AX12 | aripiprazole | 0.62 (0.39, 0.97)* | 0.91 (0.72, 1.14) | 1.47 (0.89, 2.46) |
| N05BA04 | oxazepam | 0.77 (0.61, 0.98)* | 1.03 (0.89, 1.17) | 1.32 (1.01, 1.74)* |
| N05BA12 | alprazolam | 1.41 (0.97, 2.05) | 1.36 (1.07, 1.73)* | 0.96 (0.62, 1.50) |
| N05BB01 | hydroxyzine | 0.77 (0.64, 0.93)* | 0.77 (0.69, 0.86)* | 1.00 (0.79, 1.24) |
| N05BE01 | buspirone | 1.01 (0.57, 1.76) | 0.57 (0.39, 0.83)* | 0.57 (0.29, 1.12) |
| N05CD02 | nitrazepam | 1.07 (0.66, 1.71) | 0.72 (0.52, 0.98)* | 0.67 (0.38, 1.17) |
| N05CF01 | zopiclone | 0.89 (0.73, 1.07) | 0.84 (0.74, 0.94)* | 0.94 (0.75, 1.18) |
| N05CH01 | melatonin | 0.59 (0.37, 0.90)* | 0.80 (0.64, 0.98)* | 1.36 (0.83, 2.23) |
| N05CM06 | propiomazine | 0.67 (0.55, 0.80)* | 0.79 (0.71, 0.88)* | 1.19 (0.96, 1.49) |
| N06AX11 | mirtazapine | 0.65 (0.54, 0.80)* | 0.70 (0.62, 0.81)* | 1.08 (0.85, 1.38) |
| N06BA04 | methylphenidate | 0.72 (0.47, 1.08) | 0.76 (0.59, 0.99)* | 1.06 (0.66, 1.76) |
| N06BA09 | atomoxetine | 0.47 (0.24, 0.93)* | 0.93 (0.59, 1.50) | 1.97 (0.89, 4.35) |
| N07BB01 | disulfiram | 0.55 (0.43, 0.71)* | 0.45 (0.37, 0.55)* | 0.81 (0.59, 1.11) |
| N07BB03 | acamprosate | 0.57 (0.38, 0.82)* | 0.49 (0.36, 0.66)* | 0.87 (0.54, 1.38) |
| N07BB04 | naltrexone | 0.39 (0.25, 0.58)* | 0.59 (0.43, 0.80)* | 1.51 (0.88, 2.58) |

Inclusion in table: significance of either IRR, or of ratio.

# **eMethods**

1. **Logic behind the choice of model to account for multiple testing**

The issue of multiple testing is that, as the numbers of tests increases, so do the number of chance findings that are statistically significant even in a situation where the null hypothesis is true. That is, the number of “false findings” increases as the number of tests increase. In a frequentist framework, it is not clear what tests to consider in relation to one another when adjusting for multiplicity – in theory, all tests in the world may have to be taken into account. In a Bayesian framework, by contrast, any tests that are not independent (=where either the hypotheses tested or the data used are dependent) are relevant to adjust for one another, which puts at least a theoretical limit on the extent of adjustment necessary (Sjölander and Vansteelandt 2019).^1^ A further strength of the Bayesian approach is that it allows for adjustment of effect sizes, as opposed to only significance thresholds (p-values), as in the case of the Bonferroni correction in a frequentist framework. In practice, however, the extent of adjustment necessary in even the Bayesian framework may become impractical, as the number of dependent tests may be very large. Still, this is the case for any type of practically feasible multiplicity adjustment.

In the Bayesian framework, adjustment can be done by taking into account external information about the data. In our case, the ATC subgroup of the CNS drug that is initiated reflects the type of chemical compound and the disease it is used to treat. Based on prior knowledge, we can therefore expect effect estimates within these groups to carry some information about one another. Information on the effect estimates from CNS drug initiations within these ATC subgroups can be “borrowed” from one another through the use of hierarchical modelling (Witte et al. 2000), thereby reducing the likelihood of implausible “false findings”.

1. **Mathematical description of model**

For our main analysis, we employed a two-stage Bayesian Poisson regression model, as per Witte et al. (2000),^2^ to investigate the impact of initiating a CNS drug during treatment with any SSRI or outside of any SSRI treatment. The analysis was adjusted for age, sex, and type of SSRI treatment (SSRI or no SSRI). We have used the stan_glmer function from the R package rstanarm for the hierarchical analyses.

If we were to use a conventional Poisson model for our main analyses, it could be denoted as follows:

$$\ln\left( \mu\right)= \alpha+X\underline{\beta}+W\underline{\gamma}$$

Where $\mu$ is the rate of suicidal behavior over a specified time period, $\alpha$ is the intercept term, $X$ is the matrix of unique combinations of CNS drugs and SSRI “treatments” (treatment with any SSRI or no treatment with SSRIs), $\underline{\beta}$ is the vector of regression coefficients for these combinations, $W$ is the matrix of sex, age, and SSRI treatment data, and $\underline{\gamma}$ is the vector of regression coefficients for these features.

In order to account for multiple testing, we have introduced a second-stage linear model to improve estimates of $\underline{\beta}$ by taking into account information across the CNS drug categories that are initiated. That is, we “borrow” information from the effect estimates across a CNS drug category (e.g. “N02A”) to inform the effect estimates for specific CNS drug-SSRI treatment combinations. We do this by introducing a second-stage linear regression model for $\underline{\beta}$:

$$\underline{\beta}=Z\underline{\pi}+ \underline{\delta}$$

Here, $Z$ is the matrix of the specific type of CNS drug that is initiated, $\underline{\pi}$ is the vector of coefficients associated with the specific CNS drug, and $\underline{\delta}$ is a vector of normal random variables with variances $\tau_{i}^{2}$ and mean 0 (this reflects the residual variance in $\underline{\beta}$ after taking into account the effect estimates of the individual CNS drug types).

The full two-stage model that we use in our paper can therefore be expressed as:

$$\ln\left( \mu\right)= \alpha+XZ\underline{\pi}+X\underline{\delta}+W\underline{\gamma}$$

where $\underline{\pi}$, $\underline{\gamma}$ , and $\underline{\delta}$ are all treated as random coefficients. We have used the default distributions for these in the stan_glmer function. We used similar models for the other analyses.

The following is a selection of our raw data and explanation of how it relates to the components to equations above (refer to Table A).

**Table A. Extract from data used in the main analysis**

| **Column no.** | | | | | | | |
| --- | --- | --- | --- | --- | --- | --- | --- |
|  | **1** | **2** | **3** | **4** | **5** | **6** | **7** |
| **Row no.** | **SSRI treatment (0/1)** | **CNS drug ATC group** | **CNS drug-SSRI combination** | **Person-years** | **Female (0/1)** | **Age category (years)** | **No. events** |
| 1 | 0 | N02Apre | 0-N02AA01pre | 166,3655 | 0 | 06-17 | 4 |
| 2 | 0 | N02Apre | 0-N02AA05pre | 781,1116 | 0 | 06-17 | 21 |
| 3 | 0 | N02Apos | 0-N02AA01pos | 164,0767 | 0 | 06-17 | 3 |
| 4 | 0 | N02Apos | 0-N02AA05pos | 778,4504 | 0 | 06-17 | 13 |
| 5 | 1 | N02Apre | 1-N02AA01pre | 1,232033 | 0 | 06-17 | 1 |
| 6 | 1 | N02Apre | 1-N02AA05pre | 4,032854 | 0 | 06-17 | 0 |
| 7 | 1 | N02Apos | 1-N02AA01pos | 0,977413 | 0 | 06-17 | 1 |
| 8 | 1 | N02Apos | 1-N02AA05pos | 3,811088 | 0 | 06-17 | 0 |

In Table A, the third column (“CNS drug-SSRI combination”) represents $X$. The comparison of interest is the rate of events in periods after (“pos”) versus before (“pre”) initiation of a specific CNS drug during either any or no SSRI treatment. For example, to investigate the impact of initiating N02AA01 during SSRI treatment, we compare the number of events by person-years in 1-N02AA01pos (row 7) to the number of events by person-years in 1-N02AA01pre (row 5). In order to account for the biological similarity across initiating drugs, we use information on the CNS drug ATC group (column 2), which represents $Z$, to improve the estimation of $\beta$. For example, we get the effect of initiating any CNS drug from the group “N02A” by comparing the events by person-years in any row marked “N02Apos” to the events by person-years in any row marked “N02Apre”. We can then use these estimates to “shrink” the estimates of initiating any specific CNS drug during an SSRI treatment towards each other within the CNS drug ATC groupings (in this case, within the “N02A” group). The analysis is adjusted for SSRI treatment (column 1), sex (column 5), and age category (column 6), which together represent $W$.

**References in supplementary text:**

1. Sjölander A, Vansteelandt S. Frequentist versus Bayesian approaches to multiple testing. *European journal of epidemiology* 2019;34(9):809-21.

2. Witte JS, Greenland S, Kim L-L, et al. Multilevel modeling in epidemiology with GLIMMIX. *Epidemiology* 2000;11(6):684-88.

# **Figure S1. IRRs and credible intervals of suicidal behavior from CNS drug initiation during treatment with any SSRI, by sex.**

**
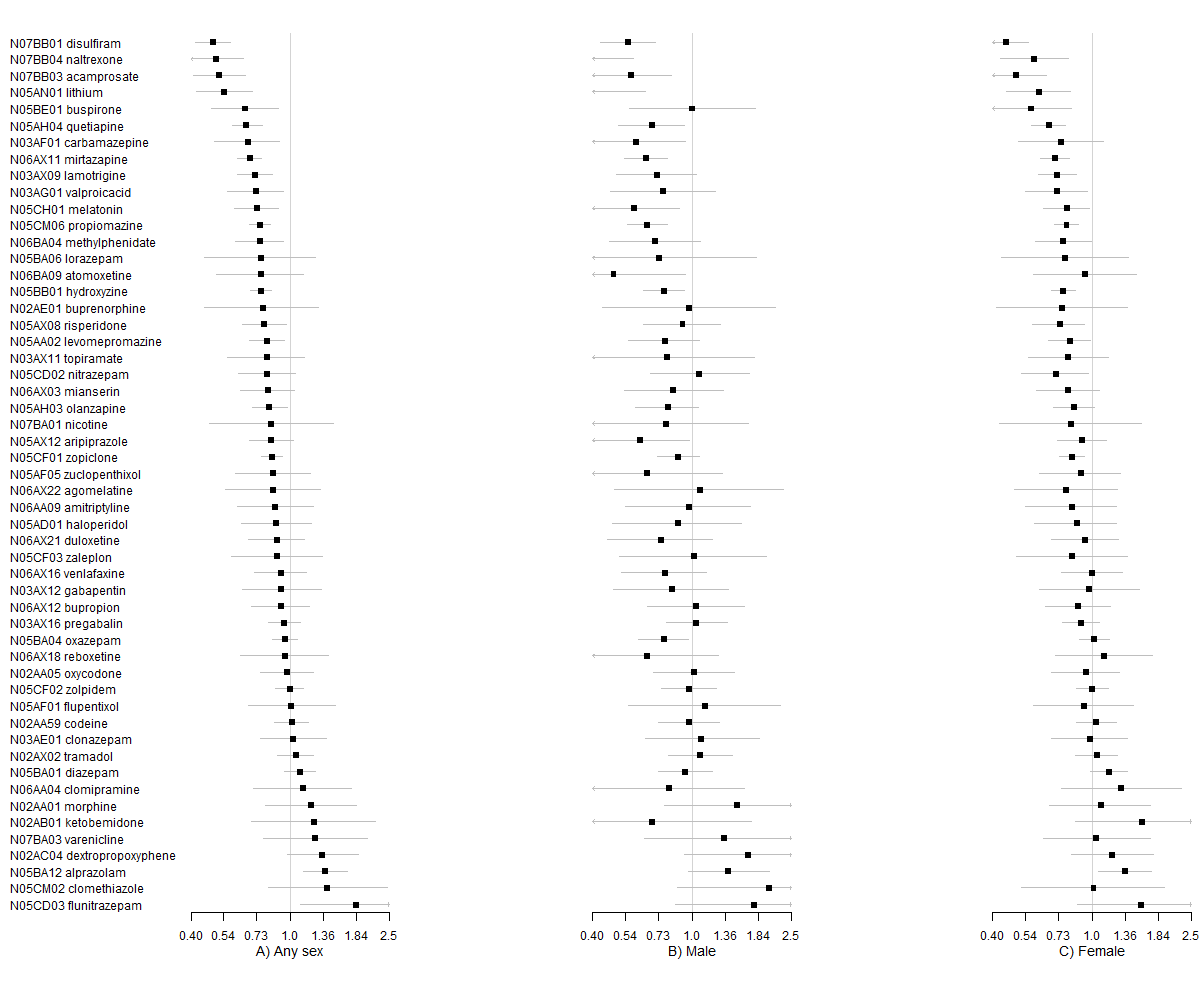
**

# **Figure S2. IRRs and credible intervals of suicidal behavior from CNS drug initiation during treatment with any SSRI and outside of SSRI treatment among individuals aged >17 years.**

**
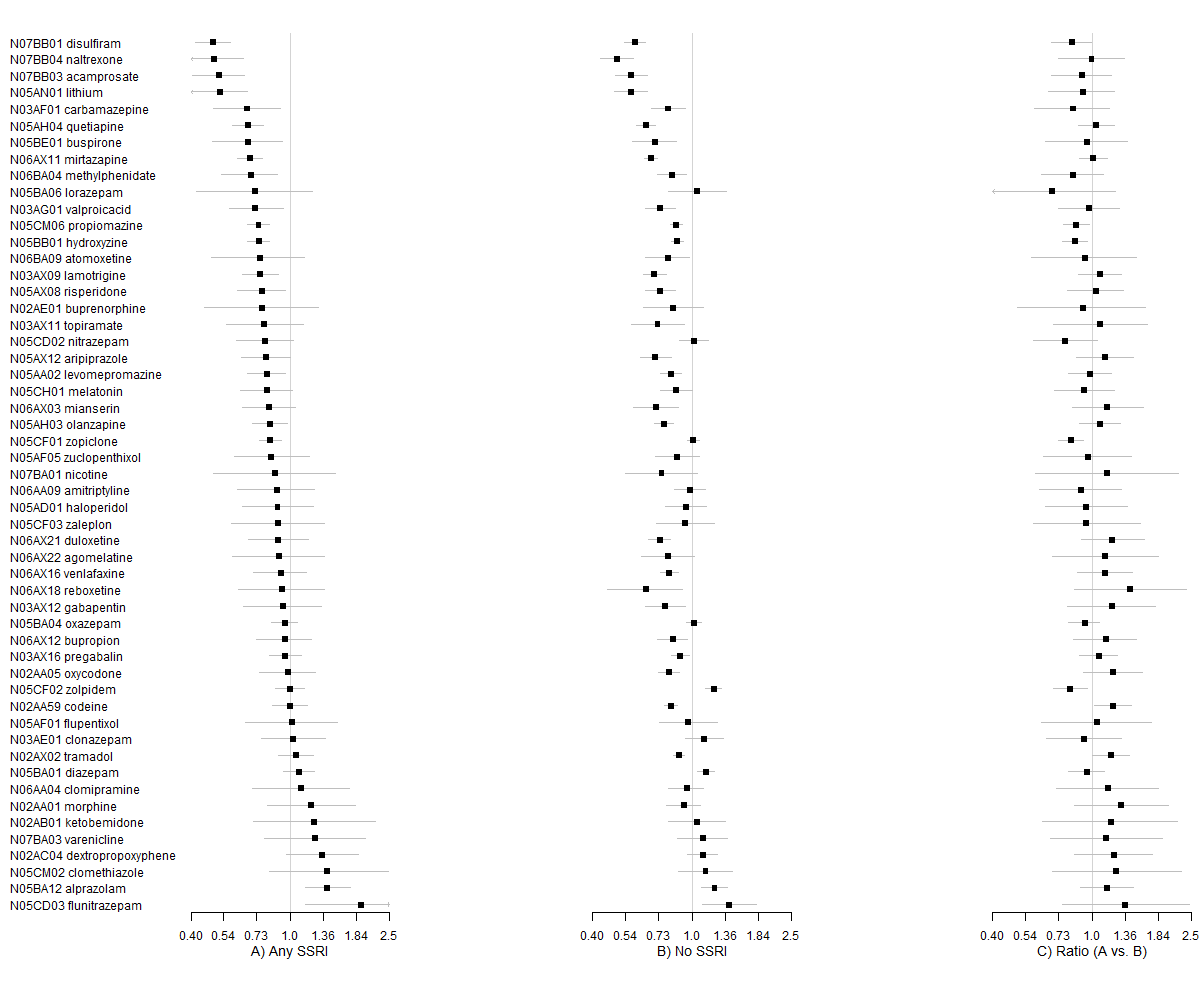
**

# **Figure S3. IRRs and credible intervals of suicidal behavior from CNS drug initiation during treatment with any SSRI and outside of SSRI treatment, considering only suicidal behavior of known intent as the outcome**

**
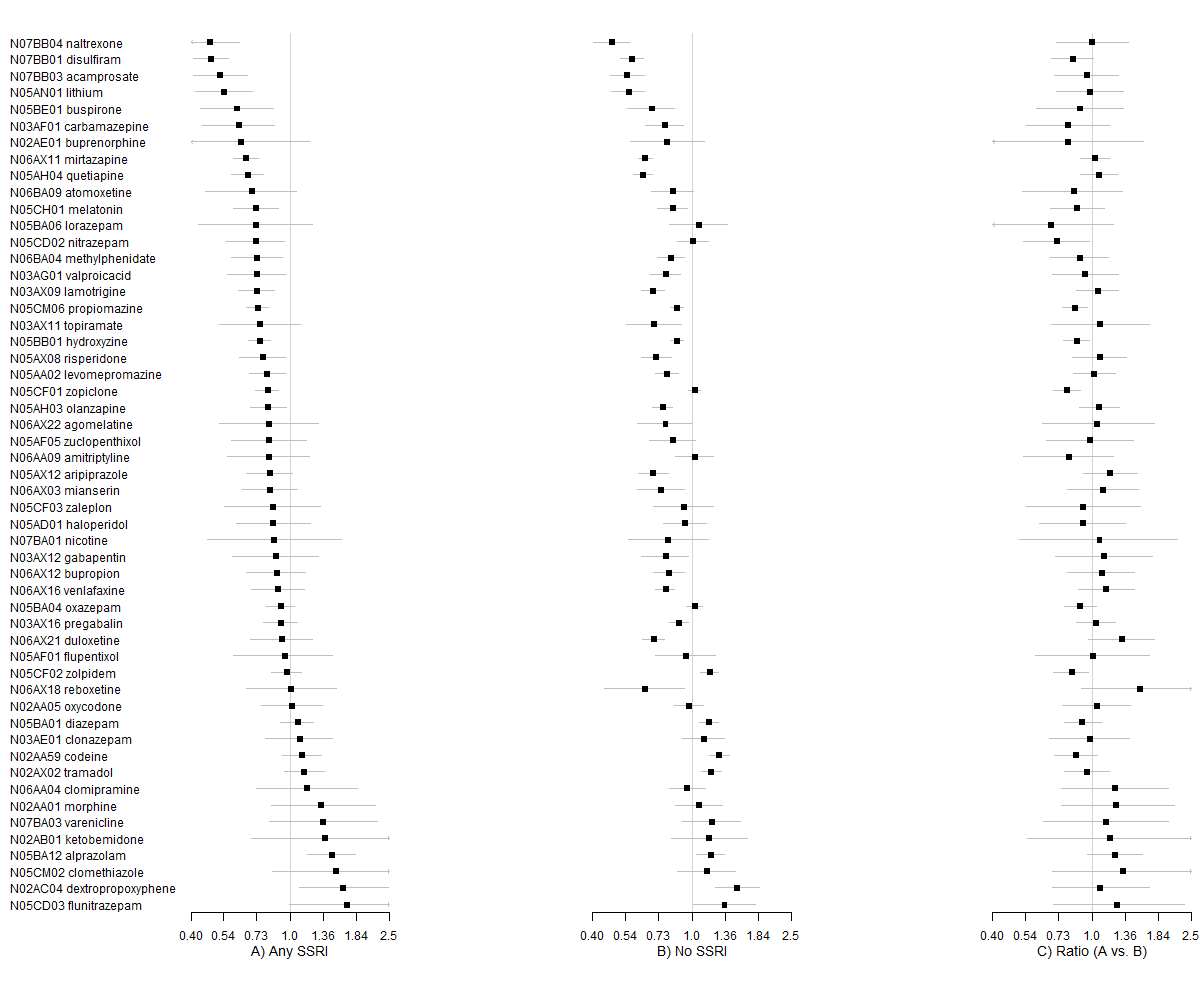
**

# **Figure S4. IRRs and credible intervals of suicidal attempts from CNS drug initiation during treatment with any SSRI and outside of SSRI treatment, when only suicide attempts are included in the outcome definition.**

**
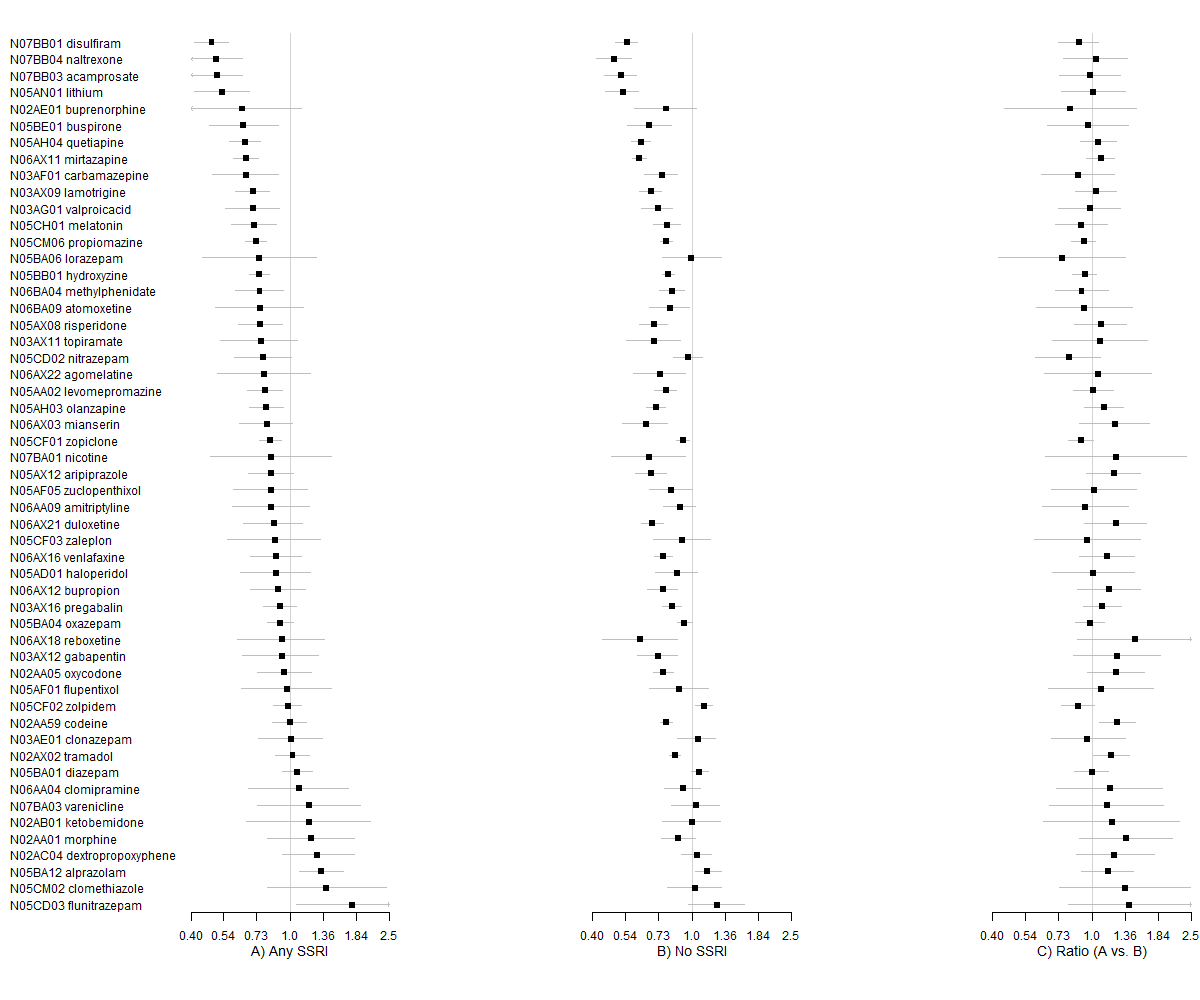
**

# **Figure S5. IRRs and credible intervals of suicidal behavior from CNS drug initiation during treatment with any SSRI and outside of SSRI treatment, excluding the date on which the additional CNS drug was initiated**

**
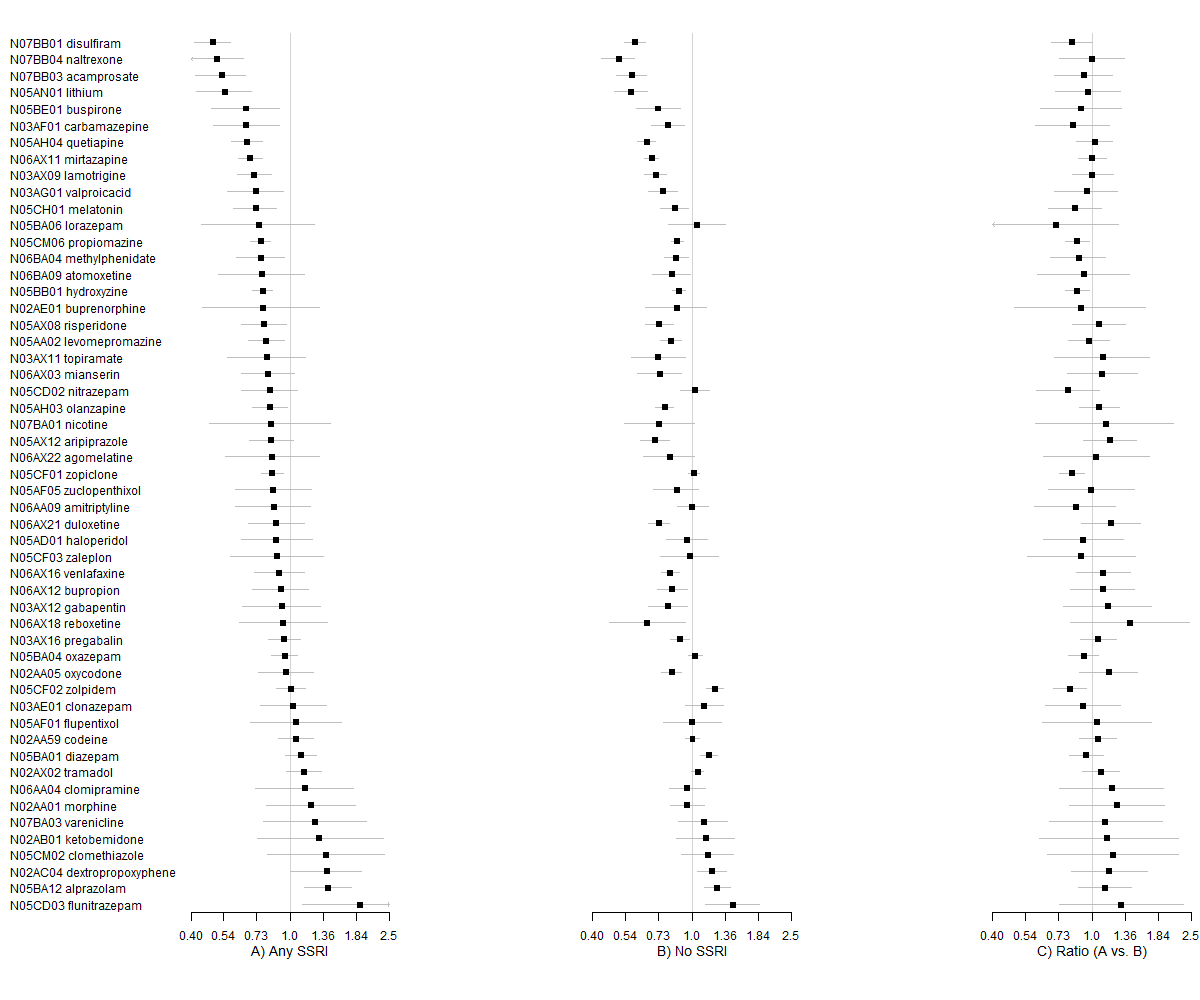
**

# **Figure S6. IRRs and credible intervals of suicidal behavior from CNS drug initiation during treatment with any SSRI and outside of SSRI treatment in the main analysis when using a frequentist Poisson regression model.**

**
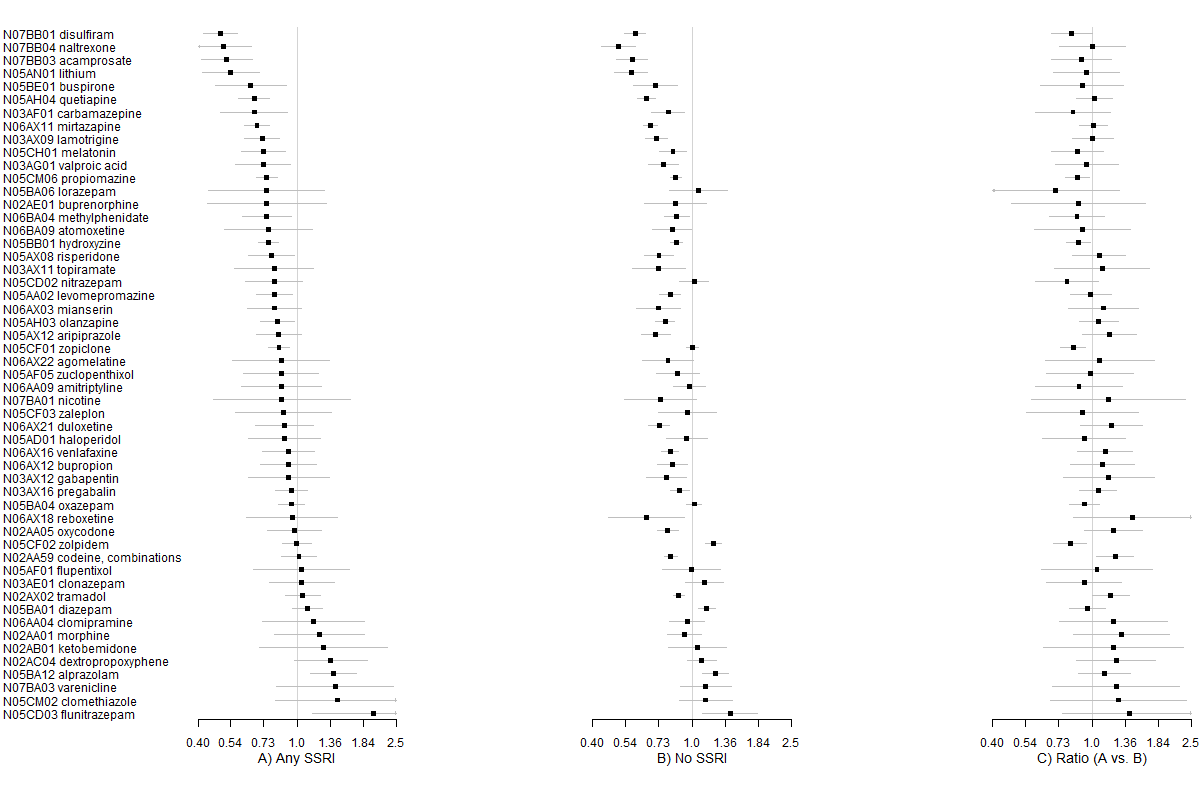
**
